# Properties of High-Swelling Native Starch Treated by Heat–Moisture Treatment with Different Holding Times and Iterations

**DOI:** 10.3390/molecules25235528

**Published:** 2020-11-25

**Authors:** Chia-Long Lin, Jheng-Hua Lin, Jia-Jing Lin, Yung-Ho Chang

**Affiliations:** 1 Department of Food and Nutrition, Providence University, Taichung 43301, Taiwan; cllin3@gm.pu.edu.tw (C.-L.L.); jiajing.lin@cgprdi.org.tw (J.-J.L.); 2 Department of Hospitality Management, MingDao University, Changhua 52345, Taiwan; jhlin@mdu.edu.tw

**Keywords:** high-swelling starch, molecular structure of amylopectin, heat–moisture treatment, holding time, iteration

## Abstract

Tapioca and potato starches were used to investigate the effect of heat–moisture treatment (HMT; 95–96 °C, 0–60 min, 1–6 iterations) on gelatinization properties, swelling power (SP), solubility and pasting properties. Tapioca starch had similar content and degree of polymerization of amylose, but a higher amylopectin short/long chain ratio, to potato starch. After HMT, the gelatinization temperature range was narrowed for tapioca starch, but was widened for potato starch. Decreases in SP and solubility were less for tapioca than potato starches, coinciding with a progressive shift to the moderate-swelling pasting profile for tapioca but a drastic change to the restricted-swelling profile for potato. Moreover, decreasing extents of SP and maximum viscosity for HMT tapioca starch were, respectively, in the range of 47–63% and 0–36%, and those of HMT potato starch were 89–92% and 63–94%. These findings indicate that the granule expansion and viscosity change of starch during gelatinization can be tailored stepwise by altering the HMT holding time and iteration.

## 1. Introduction

Starch exists as semi-crystalline granules in nature, and its properties are governed not only by its origin but also its composition, morphology, molecular characteristics and the arrangement of amylose and amylopectin within its granules [1]. Due to its instability towards heating, shearing, acidity and subsequent storage, starch is often modified to impart desired swelling properties in order to suit its application in various industrial sectors, which is particularly common for starch with a high-swelling characteristic. With respect to the modification approach, chemical modification is considered to be an effective and economical route to achieve desired outcomes, while the residual chemicals may not only raise health concerns by the public but also interfere with other key reactions in the remaining process. In contrast, heat–moisture treatment (HMT) is one of the common physical practices to modify the structure of starch with retaining its granular morphology. The resulting starch is characterized by a restricted swelling property with reduced amylose leaching, increased gelatinization temperature with broadened temperature range, and consequently, decreased pasting viscosity with enhanced granular stability [2,3,4].

Research on the HMT-resulting changes in starch architecture has revealed that some of the granules remained intact after HMT, while some were ruptured or deformed with a central void, which was accompanied by disrupted birefringence [5]. In addition to the morphological change, the HMT could alter the lamellar structure of starch in which the double helices of crystalline lamellae might not only move laterally but also vertically [6]. The lateral movement of crystallite-constituting double helices might further affect the polymorphism of starch, with the B-type polymorph being changed to a C- or A-type structure, but the A-type structure remaining unaltered [1].

Studies on the effect of HMT on properties of high-swelling starch have been carried out across a wide range of botanical origins, including tapioca [2], potato [3], sweet potato [7] and elephant foot yam [8]. Moreover, not only the moisture content and HMT temperature [1] but also the duration of heating [9] and number of iterations [10] have been elucidated in detail. Findings on the effect of HMT heating time and iteration demonstrated that the resulting decreases in swelling power and peak viscosity of starch were generally intensified by prolonging the heating time or by increasing the number of iterations. In addition, the effect of iteration tended to be more prominent than that of heating time.

A considerable amount of research has been conducted to investigate the characteristics of HMT starch, and efforts have been focused predominantly on the mechanism and HMT parameter effect on the architecture and physicochemical properties of starch, as well as HMT starch applications in food and non-food sectors. However, there is still a dearth of information on the HMT-resulting changes to the characteristics of high-swelling starch with similar amylose content but different amylopectin structure and phosphorus content, revealed by varying the HMT holding times and iterations with an aim to broaden the application of the starch. In this study, tapioca and potato starches were used as the models of high-swelling starch, and were subjected to HMT at 95–96 °C for 0–60 min with up to six iterations. Along with characterizing the molecular properties of native starch, the obtained HMT starch was analyzed for its gelatinization thermal properties, swelling power, solubility and pasting properties to investigate the effect of HMT holding time and iteration on gelatinization characteristics of high-swelling starch with similar amylose content but different molecular structure of amylopectin and phosphorus content. Moreover, the regression models on HMT-resulting changes in swelling power and pasting properties were established to elucidate the different extents of changes to the properties of starch after progressively altering its gelatinization characteristics by varying the holding time and iteration.

## 2. Results and Discussion

### 2.1. Chain Length Distribution

**Figure 1:**
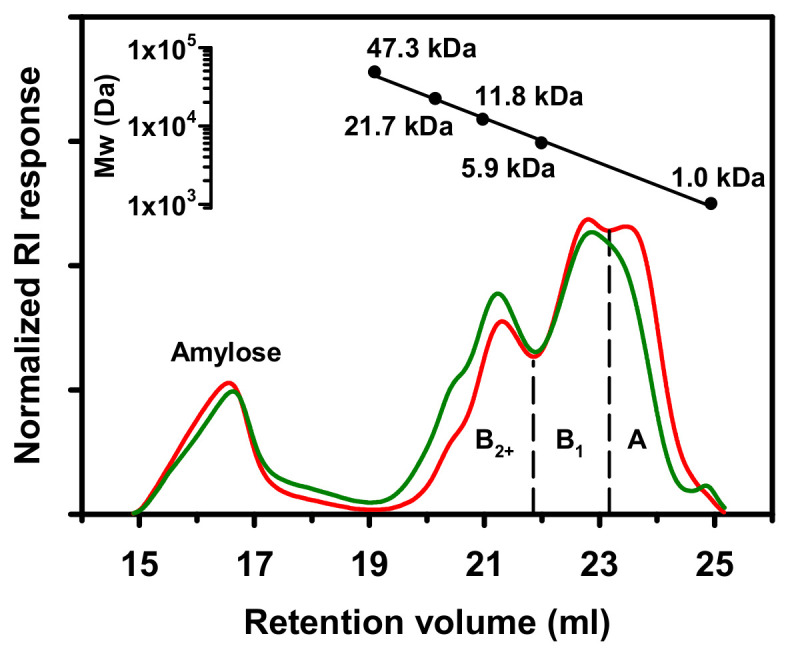
Chromatograms of isoamylase-debranched tapioca (**—**) and potato (**—**) starches.

Chain length distribution of starch used for HMT was analyzed chromatographically after being debranched by isoamylase (Figure 1). As shown in Table 1, tapioca and potato starches were of similar amylose content (~21 g per 100 g starch), and the amylose fractions were characterized with comparable weight-average degrees of polymerization (DP_w_). In contrast, the amylopectin fraction of tapioca starch consisted of more A chains but less B_2+_ chains (long chains) as compared to that of potato starch. This further reflected on the short/long chain ratio (S/L ratio) of amylopectin, with tapioca amylopectin exhibiting a higher S/L ratio than potato amylopectin. Besides, tapioca amylopectin was characterized with shorter B_2+_ chains than potato amylopectin.

The observed differences in chain length distributions of tapioca and potato starches are in agreement with the report of Srichuwong and Jane [11], in which the amylopectin molecules of A-type starch (e.g., tapioca starch) are generally characterized by shorter B_2+_ chains and a higher proportion of short chains with their branching points less localized in the inter-crystalline amorphous region when compared to those of B-type starch (e.g., potato starch). Hence, the discrepancy in amylopectin structure would probably lead to starches with different polymorphism to exhibit different thermal stability during heating.

### 2.2. Gelatinization Thermal Properties

**Figure 2:**
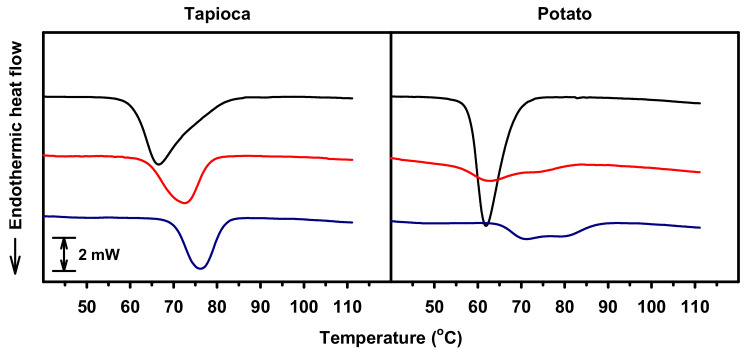
Gelatinization thermograms of native (**—**) and heat–moisture treated starches: 0-min holding time with 1 iteration (**—**) and 60-min holding time with 6 iterations (**—**).

**Figure 3:**
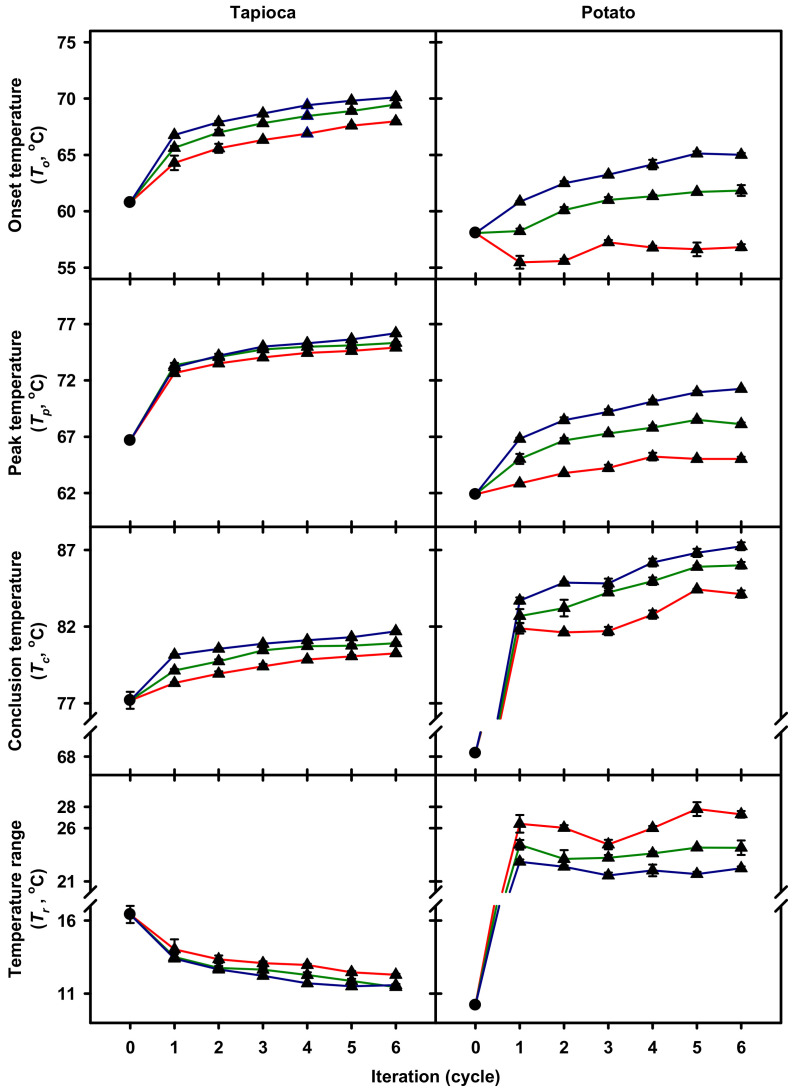
Gelatinization temperatures and temperature ranges of native (●) and heat–moisture treated (▲) starches with 0 (**—**), 30 (**—**) or 60 (**—**) min holding time and 1–6 iterations. Analyses were performed in triplicate.

Thermodynamic properties of native and HMT starches during gelatinization were analyzed calorimetrically, and the onset, peak and conclusion temperatures (*T_o_*, *T_p_*, and *T_c_*, respectively) and the corresponding enthalpy change (Δ*H*) were computed. Results showed both native tapioca and potato starches were characterized by monophasic gelatinization endotherm (Figure 2), and the *T_o_*, *T_p_* and *T_c_* were, respectively, 60.8, 66.7 and 77.2 °C for tapioca starch, and 58.1, 61.9 and 68.3 °C for potato starch (Figure 3). After HMT, tapioca starch retained the monophasic endotherm, and *T_o_*, *T_p_* and *T_c_* increased, respectively, to the range of 64.3–70.1 °C, 72.6–76.2 °C and 78.3–81.7 °C with the increasing of HMT iteration and holding time. Additionally, the HMT-resulting increase in *T_o_* was higher than that of *T_c_*, which further led to the narrowing of the gelatinization temperature range (*T_c_*–*T_o_*; *T_r_*) from 16.4 °C to the range of 11.4–14.0 °C. In contract to tapioca starch, HMT potato starch exhibited biphasic profile even with the condition of 0-min holding time and one iteration (Figure 2), and the changes in gelatinization temperatures closely depended on the condition used (Figure 3). The *T_o_* of HMT potato starch with 0-min holding time decreased to the range of 55.5–57.2 °C, while those of the starch with 30- and 60-min holding times were raised to the range of 58.2–65.1 °C. Besides, the *T_c_* of HMT starch (81.6–87.2 °C) was much higher than that of the native starch regardless of holding time. Consequently, in contrast to tapioca starch, the *T_r_* of potato starch was widened from 10.2 °C to 21.6–27.8 °C after HMT. With respect to the Δ*H* of HMT starch, the changes were relatively marginal among different HMT starches with the same botanical source [12].

In this study, it is interesting to observe that tapioca and potato starches responded differently to HMT conditions, particularly when different holding times were employed. After HMT, the increased *T_o_* was observed for all the treated tapioca starch, but was noticed for the potato starch merely with prolonged holding times (30- and 60-min). Besides, the increase in *T_c_* and decrease in Δ*H* due to HMT were considerably less for tapioca than for potato starch. Research on the HMT-resulting changes to the architecture of starch crystallites suggested that, during HMT, the crystalline region of starch was first mobilized due to the presence of moisture and the introduced thermal energy, then the constituting double helices began to move laterally and vertically [5,6]. As a result, the crystalline region progressively approached a state of improved arrangement during the process [13]. Thus, it was plausible that the mobilization of the crystalline region would commence from the region with less thermal stability. In this study, the observed differences in the effect of HMT on thermal properties of tapioca and potato starches suggest that the crystalline structure of tapioca starch appears to be more thermally stable than that of potato starch. The difference in thermal stability of these two starches could be attributed to their polymorphisms, distributions of unit chains and branching point of amylopectin molecules. Moreover, the presence of a substantial amount of phosphate monoesters, as shown by potato starch, would affect the crystalline stability of the starch to a great extent, which resulted from repelling between the phosphate groups covalently bound to starch chains [14] and further mobilizing of the crystallite-constituting double helices with less thermal stability during heating [12].

### 2.3. Swelling Power and Solubility

**Figure 4:**
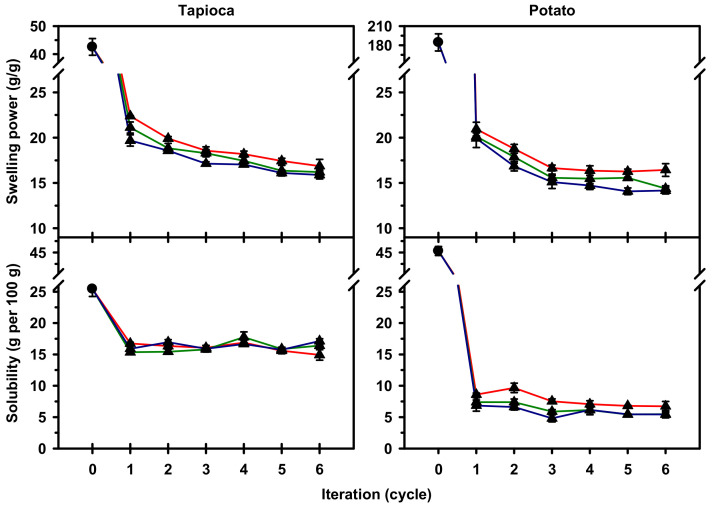
Swelling power and solubility of native (●) and heat–moisture treated (▲) starches with 0 (**—**), 30 (**—**) or 60 (**—**) min holding time and 1-6 iterations. Analyses were performed in triplicate.

Swelling power (SP) of native starch in excess of water at 90 °C was 42.6 g/g for tapioca and 184.6 g/g for potato (Figure 4). After HMT, SP of tapioca starch decreased to 15.9–22.4 g/g, and the decreasing extent was intensified with increasing iteration and holding time. For HMT potato starch, the SP decreased dramatically to 14.1–20.9 g/g, and the decrease plateaued approximately after three HMT iterations. With regard to the solubility of starch, it was observed that the solubility decreased from 25.4 to 14.9–17.7 g per 100 g for tapioca starch, and considerably from 46.0 to 4.8–9.7 g per 100 g for potato starch after HMT. Interestingly, the solubility of HMT starch with different preparation conditions was found to vary marginally with a pooled standard deviation of 0.4 g per 100 g for HMT tapioca starch and 0.5 g per 100 g for the potato starch.

It has been generally recognized that amylose molecules restrict the swelling of starch granules during gelatinization, while the amylopectin results in the opposite effect. In addition, the proximate composition of the starch, such as lipid and phosphate monoester content, affects the swelling characteristics of starch [15]. In the present study, native tapioca and potato starches were characterized with similar content and DP_w_ of amylose molecules, but had distinctly different amylopectin structure (Table 1) and phosphate monoester content [12]. Thus, it is plausible that the mentioned differences may contribute to the divergence in thermal stability between these two native starches, which leads to the observed differences in the SP and solubility at 90 °C.

Studies on the mechanism of HMT suggest that the double helices with less thermal stability are ruptured during HMT, resulting in the stabilization of the remaining crystalline structure of starch and further limiting of the swelling of granules during gelatinization [6,8]. In this study, the observed decreases in SP and solubility of HMT tapioca and potato starches were well in line with the proposed mechanism. In addition, the difference in the decreasing extent of swelling characteristics of starches after HMT suggest the thermal stability of the studied starches were substantially different, and appeared in the order of tapioca > potato starches. This result coincided with the observed calorimetric findings in which tapioca starch was more resistive to HMT than potato starch, as reflected in their HMT holding time-dependent changes in *T_o_*, increasing extent of *T_c_* and decreasing extent of Δ*H*. Moreover, the elimination of the characteristic effect of phosphate monoesters on SP of potato starch after HMT denotes that, to a certain extent, the phosphate monoesters were situated in the proximity of the double helices with less thermal stability. Apart from the mentioned changes in swelling properties, it is interesting to observe that the effect of HMT on SP and solubility of both starches remained observable at 90 °C, which exceeded the *T_c_* of HMT starches (Figure 3). This phenomenon may imply that the rupture of less-stable double helices during HMT facilitated the mobilization of the remaining double helices, resulting in the alteration of the long-range order of the crystalline structure of starch. The change in long-range structural order may further lead to the observed HMT effect on SP and solubility of the starches at 90 °C.

### 2.4. Pasting Properties

**Figure 5:**
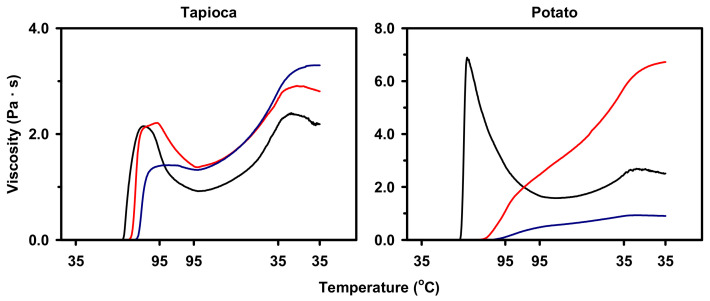
Pasting profiles of native (**—**) and heat–moisture treated starches: 0-min holding time with 1 iteration (**—**) and 60-min holding time with 6 iterations (**—**).

Pasting properties of native and HMT starches in excess water during heating and the subsequent cooling process were characterized viscometrically. Results showed that native tapioca and potato starches exhibited noticeable increases in viscosity followed by substantial paste thinning during cooking, which resembled the typical pasting profile of high-swelling starch (Figure 5). After HMT, the pasting profiles of starches were altered and the extent depended on the starch origin and the HMT condition employed. For tapioca starch with increasing iteration and holding time of HMT, the pasting profile altered progressively towards moderate-swelling profile, which was characterized with moderate peak viscosity and slight paste thinning. In contrast, potato starch after HMT exhibited a drastic change to the restricted-swelling pasting profile, which was of absent peak viscosity and continuous viscosity increase during cooling.

**Figure 6:**
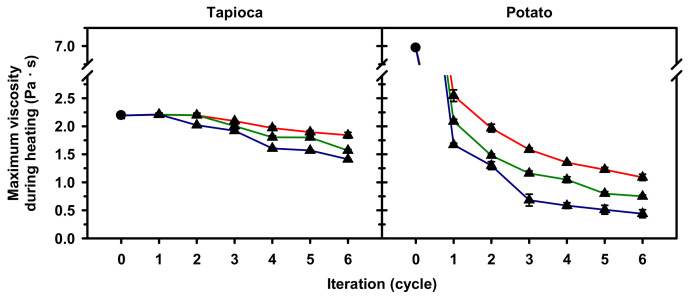
Maximum viscosity during heating of native (●) and heat–moisture treated (▲) starches with 0 (**—**), 30 (**—**) or 60 (**—**) min holding time and 1–6 iterations. Analyses were performed in triplicate.

In order to evaluate the HMT effect on gelatinization across starches with high-, moderate- and restricted-swelling pasting profiles, the maximum viscosity (*V*_max_) of starch paste, which occurred prior to cooling from 95 °C, was computed to represent the greatest viscosity of the starch that could generate under heating and with shearing force applied. As shown in Figure 6, changes in the *V*_max_ of HMT tapioca starch depended on HMT condition. With 0- and 30-min holding times, the *V*_max_ of the HMT starch began to decrease after three iterations, and the extent was intensified not only by increasing the number of iterations but also the holding time. By prolonging the holding time to 60 min, the *V*_max_ began to decrease after two iterations, and the decreasing extent was steeper than those of 0- and 30-min holding times. In contrast to tapioca starch, the *V*_max_ of potato starch considerably decreased despite the HMT condition. Additionally, a progressive decrease in *V*_max_ was observed by increasing the iteration and holding time of HMT.

Yoo et al. [15] suggested that the pasting properties of native starch were governed by its proximate composition, amylose content and unit chain profile of amylopectin. Further studies of the effect of HMT on pasting characteristics postulated that the arrangement of the crystalline structure of starch was improved after HMT, which led to the restriction of granule swelling and the consequent increase in integrity of the swollen granules against mechanical shearing during gelatinization [7,16]. Accompanying the restricted granule swelling, the leaching of amylose molecules from the granules was hampered to a certain extent, which consequently altered the network of starch matrix, as commonly reflected in the decreased peak and final viscosities of starch paste [17].

In the context of similar content and DP_w_ of amylose for tapioca and potato starches used in this study, the observation of higher *V*_max_ for native potato starch than native tapioca starch could be attributed to the high content of phosphate monoesters in potato starch, which facilitated the dispersion of starch molecules due to the negative charge repulsion between its amylopectin chains. After HMT, taking the various conditions as a whole, the observed decreases in *V*_max_ of both starches probably resulted from the strengthening of crystalline structure due to the elimination of crystallite-constituting double helices with less thermal stability. Compared to the decreasing extent of the *V*_max_ of HMT tapioca starch, the substantial decreases in those of HMT potato starch suggest that the existence of phosphate monoesters destabilizes the crystalline structure of the starch to a great extent. In addition, the B-type polymorphism and low S/L ratio of potato starch may further cause its crystalline structure to re-arrange readily, which intensifies the HMT effect on the long-range structural order of starch, consequently resulting in the manifestation of drastic changes in pasting properties.

### 2.5. Correlation between Swelling Power and Maximum Viscosity

**Figure 7:**
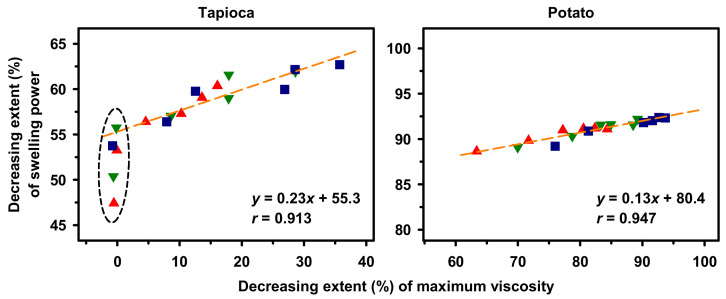
Regression of decreasing extent of swelling power on that of maximum viscosity for heat–moisture treated starches. Holding time: 0 (▲), 30 (▼) and 60 (■) min.

Regression of decreasing extents of SP on those of *V*_max_ for HMT tapioca and potato starches were computed to investigate the interplay between HMT-resulting changes in swelling and pasting properties of the native starches with high-swelling pasting profile. As shown in Figure 7, the decreasing extents of SP and *V*_max_ for HMT tapioca starch were, respectively, 47–63% and 0–36%, and the regression model appeared to be a 2-phase model. As observed for the HMT starch prepared either by 0- and 30-min holding times with 1–2 iterations or 60-min holding time with one iteration, the decreasing extent of SP ranged between 47% and 56%, while that of *V*_max_ was marginal. Thereafter, the correlation between decreasing extents of SP and *V*_max_ was positively linear (*r* = 0.913).

In contrast to HMT tapioca starch, the decreasing extents of SP and *V*_max_ for the potato starch were considerably high and in the range of 89–92% and 63–94%, respectively. Additionally, the regression model for HMT potato starch was positively linear (*r* = 0.947) throughout the HMT conditions investigated. Considering the decreasing extents and the correlations of SP and *V*_max_ for tapioca and potato starches, the observed differences suggest that the crystalline structure of tapioca starch appeared to be more stable and thus required more thermal energy to initiate its structural arrangement than that of potato starch.

## 3. Materials and Methods

### 3.1. Materials

Tapioca and potato starches were, respectively, purchased from Starpro Starch Co. Ltd. (Hangzhou, China) and Emsland-Starke GmbH (Emlichheim, Germany), and the moisture content on wet basis (wb) was 8.4–10.2%. Protein, lipid, ash and phosphorus content of starch were, respectively, 0.3 g, 0.4 g, 0.2 g and 6.5 mg per 100 g (dry basis; db) starch for the tapioca, and were 0.2 g, 0.8 g, 0.4 g and 38.8 mg per 100 g (db) starch for the potato [12]. All reagents were of analytical grade or liquid chromatography grade where appropriate.

#### Heat–Moisture Treatment

Preparation of HMT starch was performed as described by Lin et al. [12]. In brief, commercial starch was mixed with water thoroughly to adjust its moisture content to 30% (wb) then hermetically equilibrated at 4 °C overnight. A portion of starch (10 g, wb) was then hermetically heated at 95–96 °C for 0, 30 and 60 min, then cooled at 50 °C for 30 min. The aforementioned steps were performed for 1–6 iterations. Afterwards, the starch was dried at 40 °C, ground gently, and then passed through a 100-mesh screen. The moisture content of obtained starch was in the range of 9.1–11.2% (wb).

### 3.2. Methods

#### 3.2.1. Chain Length Distribution

Chain length distribution of starch was characterized in triplicate as described by Lin et al. [12] with modifications. In brief, 15 mg (db) of starch was solubilized in a solution consisting of 0.5 mL water and 4.5 mL dimethyl sulfoxide, precipitated with 15 mL of *n*-propanol, recovered by centrifugation and then re-dissolved in 1.8 mL water with the aid of heating in a boiling water bath. To the starch solution, 0.2 mL of 0.5 mol/L sodium acetate buffer and 10 μL of 50 mmol/L acetate buffer (pH 4.0) containing 0.2 μmol/min isoamylase (Megazyme E-ISAMY, from *Pseudomonas* sp., Megazyme International Ireland Ltd., Wicklow, Ireland) were pipetted sequentially and mixed thoroughly in between to lower the pH of the solution to 4.0 and to commence the debranching reaction at 40 °C for 24 h. In due course, the reaction was terminated by sequentially adding 100 μL of 0.6 mol/L NaOH, 10 μL of 1.33 mol/L (NH_4_)_2_SO_4_, 1 mL of 20 mmol/L BaCl_2_, 4.4 mL of 150 mmol/L phosphate buffer (the final pH of the solution was 6.0–6.2) and then heating in a boiling water bath for 10 min. After cooling and filtration (0.2 μm pore size), aliquot (~2 mL) of the isoamylase digest was used for high performance size exclusion chromatography (HPSEC) to analyze the chain length distribution of starch, and 5 mL of aliquot was subjected to amylose precipitation by *n*-butanol complexation. After the removal of amylose–butanol complex by centrifugation, the supernatant was subjected to HPSEC for the characterization of unit chain profile of amylopectin. The HPSEC included four series-connected gel filtration columns (one TSKgel PW_XL_, two TSKgel G3000PW_XL_ and one TSKgel G2500PW_XL_, Tosoh Corp., Tokyo, Japan), a triangle light-scattering detector (miniDAWN TREOS II, Wyatt Technology Corp., Santa Barbara, CA, USA) and a differential refractive index detector (DRI; G1362A, Agilent Technologies Inc., Santa Clara, CA, USA).

The weight percentage of amylose content was calculated as follows,

Amylose content (%) = [1 − Area_(AP)_/Area_(starch)_] *×* 100 (1)

where Area_(starch)_ and Area_(AP)_ are the areas of normalized chromatograms of debranched starch and its butanol-isolated amylopectin, respectively [12]. The DP_w_ of amylose was obtained by using ASTRA software (ver. 7.1.3, Wyatt Technology Corp., Santa Barbara, CA, USA) to compute the acquired signals of light-scattering and DRI detectors. For the amylopectin, the obtained unit chain profile was categorized into sub-fractions denoting, respectively, long B chains (B_2_ chains and longer; B_2+_ chains), B_1_ chains and A chains according to Hizukuri et al. [18]. The DP_w_ of amylopectin sub-fractions were also computed according to Lin et al. [12], and the equation for calculating the S/L ratio was as follows,

S/L ratio = (%B_1_ + %A)/%B_2+_ (2)

where %B_1_, %A and %B_2+_, respectively, denote the weight (g) of B_1_, A and B_2+_ chains per 100 g (db) starch.

#### 3.2.2. Gelatinization Thermal Properties

Gelatinization properties of starch were characterized in triplicate as described by Chang et al. [19] with modifications. In brief, 150 mg (db) starch and 450 μL water were hermetically sealed in a stainless steel crucible. After equilibrating at room temperature for 1 h with occasional inversion, the crucible was heated from 25 to 115 °C at a rate of 1.2 °C/min by using a differential scanning calorimeter (Micro DSC VII, Setaram Instrumentation, Caluire, France). The *T_o_*, *T_p_*, *T_c_* and *T_r_* of starch during gelatinization were determined. In addition, the Δ*H* was computed by integrating the corresponding endothermic heat flow after baseline correction.

#### 3.2.3. Swelling Power and Solubility

Swelling power and solubility of native and HMT starches at 90 °C were quantified gravimetrically in triplicate as described by Lin et al. [20]. In brief, starch (0.1 g, db) and water (40 mL) were transferred quantitatively into a centrifuge tube equipped with screw cap and stirring paddle, followed by incubation at 90 °C for 30 min with continuous stirring. In due course, the suspension was cooled to the room temperature and centrifuged at 4000× *g* for 15 min. The weight of sediment was recorded, and the supernatant was dried to constant weight at 130 °C to obtain the weight of soluble material.

Solubility was calculated as the mass ratio of soluble material to starch on a dry weight basis. Swelling power (SP) was expressed as the mass ratio of sediment to dry starch, which was corrected with the corresponding solubility. The decreasing extent of SP (%) was further calculated as follows,

Decreasing extent of SP (%) = (SP_native_ − SP_HMT_)/SP_native_ × 100 (3)

where SP_native_ and SP_HMT_ denote the SP of native and HMT starches, respectively.

#### 3.2.4. Pasting Properties

Pasting properties of starch during gelatinization and the subsequent cooling were characterized in triplicate with a viscometer (RVA-TecMaster, Newport Scientific Pty. Ltd., Warriewood, Australia) as described by Lin et al. [12]. Starch suspension at a concentration of 7 g (db) starch per 100 g suspension was prepared for native and HMT tapioca starches, while the concentration of 6 g (db) starch per 100 g suspension was used instead for native and HMT potato starches in order to circumvent the maximum detection limit of the viscometer. The *V*_max_ of starch paste, which occurred prior to cooling from 95 °C, was recorded for computing the decreasing extent of *V*_max_ (%) as follows,

Decreasing extent of *V*_max_ (%) = (*V*_max-native_ − *V*_max-HMT_)/*V*_max-native_ × 100 (4)

where *V*_max-native_ and *V*_max-HMT_ denote the *V*_max_ of native and HMT starches, respectively.

#### 3.2.5. Statistical Analysis

Correlation between decreasing extents of SP and *V*_max_ of starch after HMT was computed by using the first-order regression model. The Pearson correlation coefficients (*r*) were obtained subsequently.

## 4. Conclusions

Tapioca and potato starches, having similar properties of amylose molecules but differing in chain length distribution of amylopectin, were used as the models to investigate the effect of HMT on the properties of high-swelling starch from different origins. After HMT, tapioca starch was characterized with increased gelatinization temperatures and narrowed *T_r_*, and the changes were further intensified by increasing the number of iterations and prolonging the holding time. Similar changes were also observed for HMT potato starch, while a decrease in *T_o_* was noticed for the starch with 0-min holding time. Besides, the *T_r_* of HMT potato starch was found to be widened across all the HMT conditions applied.

The different extents of HMT-resulting effect were also observed in the magnitudes of decreases in SP and the transformation of the pasting profile, where a transformation from high-swelling to moderate-swelling profile was observed for HMT tapioca starch, but a drastic change to restricted-swelling profile was observed for the potato starch. The differences in HMT effect on swelling and pasting characteristics also reflected on the regression models on decreasing extents of SP and *V*_max_ of the HMT starches.

Results of this study indicate that the thermal stability, granule expansion and viscosity change of high-swelling starch during gelatinization, as well as the viscosity of starch paste during the subsequent cooling process, can be tailored in a stepwise manner by altering the HMT holding time and iteration. Moreover, the findings of the study suggest that the dissimilarities in S/L ratio of amylopectin and phosphate monoester content of the starting starches probably result in the divergence in thermal stability of their crystalline structure, leading to the observed differences in the extents of the HMT-resulting effect. The low S/L ratio and high phosphate monoester content, as shown by potato starch, may likely further intensify the HMT effect on the decreases in swelling and pasting characteristics of the obtained starch. Additionally, the observation of synergistic effect of phosphate monoesters and HMT on starch properties may demonstrate an alternative approach for imparting both features of low swelling and retrogradation to a starch.

## Figures and Tables

**Table 1:** Chain length distribution parameters of starch ^1^_._

	Tapioca	Potato
Amylose		
% ^2^	21.3 ± 0.5	21.2 ± 0.3
DP_w_^3^	9944 ± 925	9234 ± 123
Amylopectin		
B_2+_ chains		
%	21.3 ± 0.4	29.6 ± 0.2
DP_w_	57.8 ± 1.4	63.1 ± 0.2
B_1_ chains		
%	29.9 ± 0.5	27.6 ± 0.0
DP_w_	23.4 ± 0.1	23.7 ± 0.0
A chains		
%	27.4 ± 0.9	21.6 ± 0.2
DP_w_	11.6 ± 0.1	12.4 ± 0.0
S/L ratio ^4^	2.69 ± 0.08	1.66 ± 0.01

^1^ Data are expressed as the mean values (*n* = 3) ± standard deviation. ^2^ %: weight (g) of the corresponding fraction per 100 g (db) starch. ^3^ DP_w_: weight-average degree of polymerization expressed in the unit of anhydrous glucose unit. ^4^ Short/long chain ratio = (%B_1_ + %A)/%B_2+_.
